# Liquid Chromatographic Enantioseparations Utilizing Chiral Stationary Phases Based on Crown Ethers and Cyclofructans

**DOI:** 10.3390/molecules26154648

**Published:** 2021-07-31

**Authors:** Róbert Berkecz, Gábor Németi, Antal Péter, István Ilisz

**Affiliations:** Interdisciplinary Excellence Centre, Institute of Pharmaceutical Analysis, University of Szeged, Somogyi u. 4, H-6720 Szeged, Hungary; berkecz.robert@szte.hu (R.B.); nemeti.gab@gmail.com (G.N.); apeter@chem.u-szeged.hu (A.P.)

**Keywords:** liquid chromatography, chiral stationary phases, enantiomeric separations, crown ethers, cyclofructans

## Abstract

Natural compounds can exist in different forms, where molecules possessing chirality play an essential role in living organisms. Currently, one of the most important tasks of modern analytical chemistry is the enantioseparation of chiral compounds, in particular, the enantiomers of compounds having biological and/or pharmaceutical activity. Whether the task is to analyze environmental or food samples or to develop an assay for drug control, well-reproducible, highly sensitive, stereoselective, and robust methods are required. High-performance liquid chromatography best meets these conditions. Nevertheless, in many cases, gas chromatography, supercritical fluid chromatography, or capillary electrophoresis can also offer a suitable solution. Amino acids, proteins, cyclodextrins, derivatized polysaccharides, macrocyclic glycopeptides, and ion exchangers can serve as efficient selectors in liquid chromatography, and they are quite frequently applied and reviewed. Crown ethers and cyclofructans possessing similar structural characteristics and selectivity in the enantiodiscrimination of different amine compounds are discussed less frequently. This review collects information on enantioseparations achieved recently with the use of chiral stationary phases based on crown ethers or cyclofructans, focusing on liquid chromatographic applications.

## 1. Introduction

As living systems often display different responses to the enantiomers of chiral compounds such as drugs, agrochemicals, food additives, and fragrance materials, strong demand has arisen in the life and pharmaceutical sciences for analytical tools to separate individual stereoisomers. Because of their identical physical and chemical properties, the separation of enantiomers requires a chiral environment. Under these conditions, the enantiomers of the sample form diastereomeric pairs with the so-called selector, which provides the stereospecific chemical interactions required for separation. In the case of the so-called indirect separations, the reaction of enantiomers with a homochiral reagent (selector) results in the formation of diastereomeric derivatives that could be separated on achiral columns. Indirect methods were first to provide effective enantioseparations in the field of chiral analysis. However, over the years, direct methods applying a selector either dissolved in the mobile phase or bound to a support material have become more popular. Following the introduction of chiral stationary phases (CSPs) where the selector is immobilized on a solid support, the use of a selector dissolved in the mobile phase has lost its practical importance in high-performance liquid chromatography (HPLC). The main field of application for the dissolved selector is currently capillary electrophoresis.

Nowadays, in the case of chiral selectors covalently attached to silica gel, the chromatographic separation is based on the temporary formation of diastereomeric pairs through the interactions between the enantiomers to be separated and the selector. The reactions that reversibly form diastereomeric pairs on the surface of the stationary phase are illustrated in Equations (1) and (2), where (*R*)-SO stands for a selector with *R* configuration, *K_S_* and *K_R_* indicate the equilibrium constant of the diastereomeric complexation reaction between the selector and the enantiomer with *S* or *R* configuration, respectively, while (*R*)-E and (*S*)-E denote the enantiomer with *R* or *S* configuration, respectively.

*K_S_* (*R*)-SO + (*S*)-E ⇌ [(*R*)-SO --- (*S*)-E] (1)

*K_R_* (*R*)-SO + (*R*)-E ⇌ [(*R*)-SO --- (*R*)-E] (2)

The different retention behaviors of the enantiomers can be attributed to the difference in the equilibrium constants of the reactions, leading to the formation of the diastereomers. Chiral separations are accomplished through the formation of noncovalent interactions, similar to in an achiral chromatographic system. As the mobile phase components can influence the structure and solvation of both the stationary phase and the enantiomers to be separated, by the variation in mobile phase composition, the nature and strength of the interactions can be modified. As a result, the equilibrium constants (*K_S_*, *K_R_*) can be varied and, consequently, enantiomeric separations can be affected.

The first chiral stationary phase was described in 1966, when enantiomers of α-amino acid derivatives were separated on a capillary column by gas chromatography [1]. A few years later, Davankov and Rogozhin introduced chiral ligand-exchange chromatography and, thus, the first LC applications appeared [2]. Liquid chromatographic innovations in the 1970s and 1980s led to the intensive development of HPLC equipment and packed columns. The appearance of mechanically stable, porous silica particles of small diameter led to the commercialization of a new family of CSPs with high efficiency. By the end of the 1990s, more than 200 stationary phases had entered the market [3], and the number of CSPs has continued to increase further ever since in line with increasing demand.

In principle, “any” chiral molecule can be considered a stationary phase selector, yet they tend to be derived from relatively few classes of compounds. These include amino acids, peptides, oligosaccharides (cyclodextrins and cyclofructans), polysaccharides (derivatized cellulose or amylose), macrocyclic compounds (antibiotics and crown ethers), molecules providing donor–acceptor interactions (Pirkle-type selectors), ion exchangers, and other selectors with limited importance (synthetic polymers, molecularly imprinted polymers, chiral ionic liquids, etc.). The chiral selectors utilized as CSPs have been reviewed and discussed in detail recently [4,5,6,7,8,9,10]. The most frequently applied selectors and their important interactions are summarized in Table 1.

Chiral separation techniques have become a very sophisticated field of analytical chemistry by now. The continuous interest is indicated by numerous scientific publications describing new CSPs and their applications. Although still at the research and development level, superficially porous (SP) sub-3 µm and fully porous (FP) sub-2 µm particles with narrow size distributions have already been utilized for the preparation of CSPs with ultrahigh efficiency [11,12,13]. It can be expected that the further development of novel CSPs and related “chiral columns” will be of high interest, and extremely fast enantioseparations with ultrahigh efficiency will be available for routine analyses in the near future.

**Figure 1:**
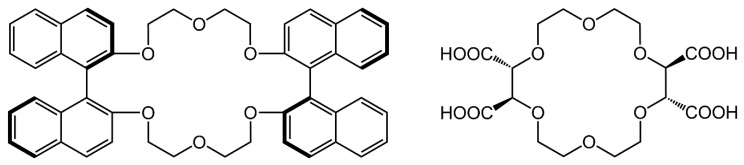
The structures of (*R,R*)-bis-(1,1′-binaphthyl)-22-crown-6 (**left**) and (*+*)-(18-crown-6)-2,3,11,12-tetracarboxylic acid (**right**).

**Figure 2:**
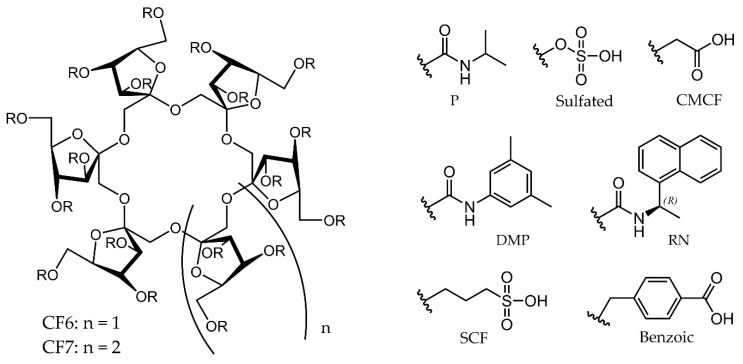
The general structure of cyclofructans (**left**) and their derivatives (**right**).

Crown ethers and cyclofructans of the numerous selectors utilized for enantioseparations at present are discussed less frequently. These two different classes of compounds possess somewhat similar structural peculiarities, as can be expected on their structures (Figure 1 and Figure 2). Consequently, they can be characterized with a similar recognition mechanism, and both of them can be applied for the separation of enantiomers containing a primary amino group. In this review, we summarize the results with respect to CSPs based on crown ethers and cyclofructans reported between 2015 and the first half of 2021, focusing on liquid chromatographic applications.

## 2. Crown Ether-Based Selectors

Polyethers belonging to the family of macrocyclic compounds can form complexes with alkali- and alkaline-earth-metal ions and ammonium ions. In these cases, the central ion is placed into the ring of the polyether “crown” [14]. In general, the oxygen atoms can serve as electron donors, while the cavity allows inclusion complexation of compounds of a specific size. Utilizing this property, crown ether-based CSPs are best suited for the separation of compounds containing primary amino groups of similar size to alkali- or alkaline-earth-metal ions. The alkyl- or aryl-ammonium ion formed from the amino group under acidic conditions is attached to the crown ether through the formation of an inclusion complex. Additional interactions contributing to the chiral recognition might originate from steric factors of the substituents of the chiral ammonium ions and the residues attached to the chiral moieties incorporated into the crown ether.

In 1979, Sogah and Cram were the first to report enantioselective separation on a crown ether-based CSP bound to a polystyrene skeleton [15]. About two decades later, Hyun and coworkers [16] and Machida et al. [17] described the synthesis and use of stationary phases based on chemically bound crown ether to separate enantiomers of compounds containing a primary amino group. Several crown ethers have been studied in recent decades; however, two types of chiral crown ethers have been utilized efficiently for the preparation of CSPs. One is derived from bis-(1,1′-binaphthyl)-22-crown-6, and the other from (18-crown-6)-2,3,11,12-tetracarboxylic acid (18-C-6-TA) [18,19,20,21]. Their structures are presented in Figure 1.

In addition to the support, the functional groups of the selector and/or the linker (spacer) may significantly affect chromatographic properties and chiral discrimination. It is valid for all crown ether-based stationary phases that the formation of the ammonium ion–crown ether complex is essential, but it is not necessarily a sufficient criterion for chiral recognition. For more details, the reader is referred to review articles published earlier [18,19,20,21].

## 3. Selectors Based on Cyclofructans

The best-known members of the family of macrocyclic oligosaccharides are cyclodextrins, which play a prominent role in chiral separation techniques. Cyclofructans (CFs) also belong to this family, but they differ significantly in their structure and behavior from cyclodextrins. CFs are composed of six or more *β*-2,1-linked d-fructofuranose subunits. Their general structure and applied derivatives are shown in Figure 2.

Among cyclofructans, the so-called CF6 member, containing six fructofuranose units, is of greater importance, because its pure form is easily accessible and its geometry is well defined [22]. It is important to note that CF6, unlike cyclodextrins, does not have a central hydrophobic cavity. Accordingly, it is not able to form a hydrophobic inclusion complex. Unmodified CF6 bears six fructofuranose units each containing four centers of asymmetry and three OH groups. The central core shows the same structure as the corresponding crown ether and this is the reason why its chromatographic behavior resembles that of the crown ethers. Similar to crown ethers, the interaction between the protonated primary amino group and the core created by oxygen atoms are decisive for enantioseparation. Obviously, the modification of the selector provides an opportunity to increase efficiency and change selectivity.

Enantioselective separations take place with different separation mechanisms for CF6-based stationary phases modified with different substituents. The structure of aliphatic CF6 with minimal functionalization has a “loose,” open central portion. In contrast, CF6 containing aromatic substituents with a higher degree of substitution has a significantly more “crowded” ring structure. It prevents access to the inside of the selector but, at the same time, ensures different interactions on the outside. The development of CF-based columns can be attributed to the Armstrong group. Their first paper was published in 2009 reporting the separation of primary amines on natural and modified CF6 stationary phases [23]. More detailed discussion on the retention mechanism can be found in related references [24,25].

## 4. Recent Applications of Crown Ether-Based CSPs

The constantly growing demand for the enantiomeric separation of new chiral compounds has initiated the preparation of new types of CSPs, for instance, crown ether-based CSPs, and the development of related analytical methods. Since 2015, twenty significant scientific papers have been reported in this relatively narrow segment of direct chiral chromatography. Related main pieces of information are summarized in Table 2.

**Figure 3:**
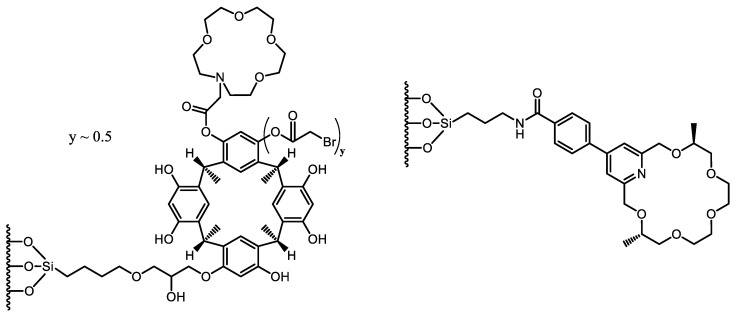
Structures of CSPs applied in Ref. [26] (**left**) and Ref. [27] (**right**).

The newly synthesized aza-15-crown-5-capped (3-(C-methylcalix [4] resorcinarene)-2-hydroxypropoxy)-propylsilyl-bonded silica particles (15C5-MCR-HPS), packed into a 150 mm × 2.0 mm I.D. stainless-steel column, were successfully applied in the separation of positional isomers of disubstituted benzenes (nitroanilines and nitrophenols) and enantiomers of chiral drug compounds in HPLC under NP, RP, and PO conditions [26]. The anchored (3-(C-methylcalix [4] resorcinarene)-2-hydroxypropoxy)-propyltrimethoxysilane as a chiral selector with aza-15-crown-5 moieties (Figure 3) forms multiple interactions, providing excellent and robust separation of the enantiomers of chiral drug compounds, e.g., promethazine (*R_S_*: 7.74), 1-phenyl-1-propanol (*R_S_*: 5.78), warfarin (*R_S_*: 2.03), and α-methylbenzylamine (*R_S_*: 6.83), under RP conditions.

The presence of the pyridine ring substituted with an aromatic linking unit on the 18-crown-6 ether-based chiral selector may provide additional π–π interactions with aromatic groups of the guest molecules and enhance enantiomeric recognition accordingly. Protonated primary arylalkylamines and perchlorate salts of α-amino acid esters were selected for studying the enantioseparation properties of a new pyridino-18-crown-6 ether-based CSP (Figure 3) under different PI mobile phase compositions [27]. The enantiomeric separation ability of the crown ether selector could be additionally changed by inserting an acridine tricyclic unit or its derivatives, making the crown ring more rigid.

**Figure 4:**
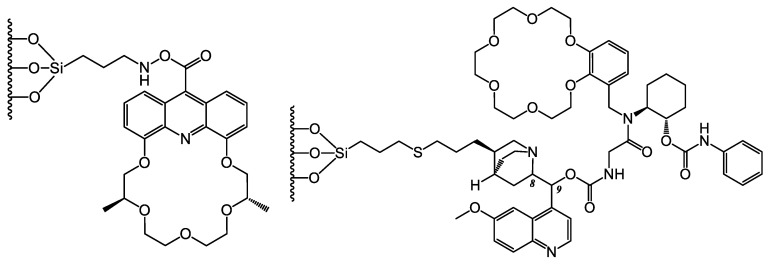
Structures of CSPs applied in Ref. [28] (**left**) and Ref. [29] (**right**).

Among several newly synthesized 18-crown-6 and 21-crown-7 ether derivatives, one was selected for the preparation of a new CSP (Figure 4, left) to study its role in the enantiomeric separation of arylalkylamines and α-amino acid esters [28]. The results underlined the importance of the distance of the chiral center from the acridine ring, which was found to be decisive for the efficiency of enantiomeric recognition.

Another study also drew attention to the essential role of the chiral environment in the crown ether ring [29]. Adding an additional (1*S*,2*S*)-2-aminocyclohexyl phenylcarbamate (Heca) chiral group to the *Cinchona* alkaloid crown ether chiral selector (Figure 4, right) resulted in improved chiral recognition of the primary amine enantiomers. However, the modification of the Heca ion-exchange site was found to be unfavorable in the separation of chiral acids. A change in the absolute configuration of the chiral selector resulted in a change in the elution order for acidic compounds, while no effect was found for primary amines and amino acids.

**Figure 5:**
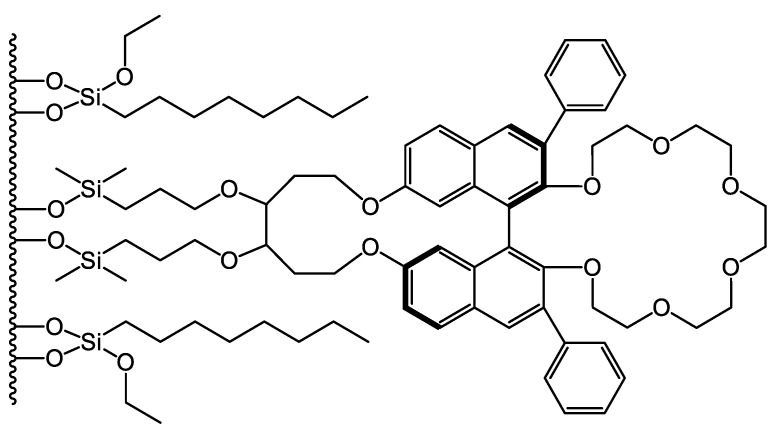
Structure of CSP applied in Ref. [30].

Protection of the residual silanol groups of (3,3′-diphenyl-1,1′-binaphthyl)-20-crown-6 (Figure 5) with *n*-octyl groups assisted in improving the chiral recognition of valacyclovir and its analogs through decreasing nonenantioselective interactions [30]. This kind of silanol surface modification also influenced the retention behavior of the investigated compounds, which can be attributed to more pronounced lipophilic interactions due to the *n*-octyl groups. Both the type and the concentration of acidic modifiers and the organic content of the aqueous mobile phase were crucial in the retention and enantiomeric separation of interested analytes.

The chiral analytical methods using hyphenated techniques, such as HPLC coupled to a mass spectrometer (HPLC-MS), provide an opportunity to obtain enhanced sensitivity without the need for the derivatization of chiral analytes. However, during MS detection, the ionization efficiency of enantiomers is determined by the mobile phase composition. Thus, constant eluent composition is an important issue for the quantitative determination of enantiomeric purity. A relatively fast isocratic RP HPLC-MS method was developed for chiral separations of underivatized α-amino acids on a CROWNPAK CR-I (+) column (Daicel, Corp., Osaka, Japan) [31]. In a subsequent report of the authors, the LC-MS method was further developed and applied for the analysis of amino acids in black vinegar [32]. In a third study, they applied a 50 × 3.0 mm CROWNPAK CR-I (+) column packed with 3 µm particles instead of a 150 × 3.0 mm column with 5 µm particles. Modifying the original mobile phase composition and flow rate, and reducing the extra-column band broadening, baseline separation was achieved within 2 min for 18 pairs of proteinogenic amino acids [33]. The modification of eluent composition provided baseline separation within 45 s for amino acid enantiomers except for DL-His, DL-Leu, and DL-Ile.

As a demonstration for the achiral RP separation of substituted aniline position isomers, CROWNPAK CR (−) and CR (+) columns were utilized [34]. Quantum chemical calculations were performed to understand the achiral interactions occurring during the recognition process, where a two-point binding mechanism through two hydrogen bonds between the ammonium ion of substituted anilines and the two oxygen atoms of the crown ether ring was suggested. The calculation was extended for aggregates of phenylglycine enantiomers and the crown ether selector, and the results were well correlated with the obtained chromatographic elution order. Interestingly, a two-point binding model was predicted for the *S* enantiomer, while a three-point binding chiral mechanism was found for the *R* enantiomer of phenylglycine.

Applications of CROWNPAK CR-I (+) and CR-I (−) columns for the separation of d-amino acid-containing antimicrobial tripeptide diastereomers were reported [35]. Based on the performed molecular dynamics (MD) simulations, three hydrogen bonds between the *N*-terminal amino group (primary ammonium ion) and oxygens in the crown ether core were found to be the dominant interactions. The contribution of hydrophobic interactions between the phenyl rings of the selector and the peptides was also suggested. Free side amino groups containing tetrapeptide (Tyr-Arg-Phe-Lys-NH_2_) diastereomers were successfully separated on CROWNPAK CR-I (+) and Chirosil RCA (+) (Regis Technologies Inc., Morton Grove, IL, USA) columns under isocratic RP elution mode [36]. Similar to earlier studies, the application of perchloric acid afforded better separation of the investigated peptides than other acid additives, and a higher acid concentration–higher retention relation was observed due to enhanced protonation of the amino groups at lower pH of the mobile phase. The change in acetonitrile content in the binary acetonitrile–water mobile phase system resulted in U-shaped retention plots, which were accounted for by the shifting balance between hydrophilic and hydrophobic separation mechanisms. As a potential pharmaceutical application of a crown ether column, a validated analytical method was published for the quantitative determination of the *S* enantiomer of dihydropyridine calcium channel blocker amlodipine in tablet formulations containing racemic amlodipine [37]. The enantiomer separation of amlodipine was performed on the CROWNPAK CR (+) column under isocratic RP mode.

**Figure 6:**
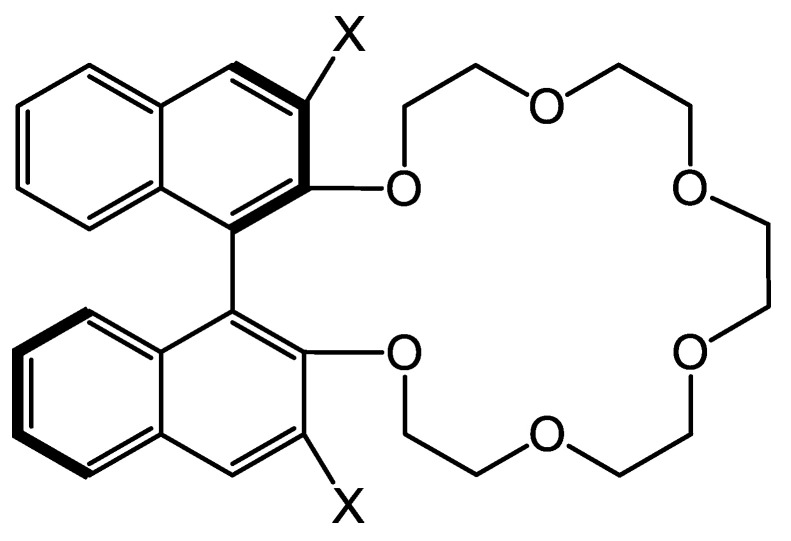
Structure of CSPs applied in Ref. [38] (X: Cl, Br, or I).

To improve the chiral recognition ability of the CROWNPAK CR (+) selector, three *R*-(3,3′-X-substituted-1,1′-binaphthyl)-20-crown-6 (X: Cl, Br or I) selectors (Figure 6) containing CSPs were synthesized and compared in the enantiomeric separation of α-amino acids [38].

For the investigated amino acids, Br-substituted CSP had the highest chiral recognition ability and it provided a better resolution than the commercially available CSP. For the development and study of the direct enantiomeric separation of alogliptin, linagliptin, and saxagliptin, CROWNPAK CR-I (+) CSP was selected regarding the free amino group content of the investigated dipeptidyl peptidase-4 inhibitors [39]. Method optimization was performed by studying the effects of the solvent, additive, pH, and column temperature.

As an efficient, fast analytical technique, supercritical fluid chromatography (SFC) has been widespread in chiral chromatography, and it was successfully applied in the crown ether-based HPLC-MS separation of underivatized α-amino acids enantiomers [40]. All 18 selected amino acid racemates, except histidine, were separated within 3 min, and the obtained mean *R_S_* value was higher than 5. Similar to HPLC separation, the D enantiomer was first eluted in all cases. A biological application of crown ether-based chiral separation of urinary quinolone racemates such as flumequine, primaquine, lomefloxacin, tafenoquine, and ofloxacin was performed [41]. The developed method was validated and thermodynamically characterized, and an enthalpy-controlled separation mechanism was described.

Nowadays, UHPLC–tandem mass spectrometry methods (UHPLC–MS/MS) play an important role in targeted analysis of endo- and exogenous compounds in pharmaceutical and biological samples. For the determination of the enantiomeric purity of levothyroxine sodium tablets containing the thyroxine hormone, the UHPLC–MS/MS method based on chiral separation on ChiroSil RCA (+) CSP was developed and validated [42].

**Figure 7:**
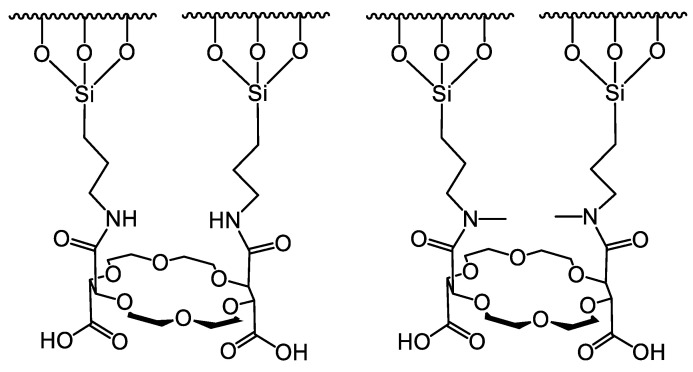
Structures of CSPs applied in Refs. [43,44].

In two comparative studies, two types of CSPs based on (+)-(18-crown-6)-2,3,11,12-tetracarboxylic acid were examined in the chiral separation of cyclic α-amino acids, including proline, pipecolic acid, and their derivatives [43], as well as methoxyphenamine and its analogs [44]. The CSPs differed only in the methylation of amide groups of the selector; the original selector has primary amides on the linkers, while the other has secondary amide groups (Figure 7). Overall, the use of the CSP containing more substituted amide linkers proved to be better in the chiral recognition of the investigated compounds. A possible explanation is that the methylated amide group cannot form an intermolecular hydrogen bond with two intramolecular hydrogen bonds with the ether oxygens of the crown ether ring as in another selector. Therefore, these unhidden ether oxygens can be involved in complex formation with the ammonium ion of the examined chiral compound.

## 5. Recent Applications of Cyclofructan-Based CSPs

Since their introduction by Armstrong et al. [23], several research papers have described the utilization of CF-based CSPs. Results published since 2015 are summarized in Table 3 and discussed below.

CSPs were designed to bear positive charges with protonated imidazolium, pyridinium, or ammonium groups, and they were tested with a set of 34 chiral analytes including acids, bases, and neutral compounds applying nine different eluent systems [45]. The best enantioselectivities were achieved in NP mode, while none of the compounds could be separated under RP conditions. It was confirmed that the bulkiness of the substituent group can be a major hindrance in chiral recognition by CF6-based CSPs, and a degree of substitution of the CF6 greater than six was found to be detrimental to the enantiorecognition ability of the selector. Better-than-baseline separation was achieved for 20 of 21 chiral ruthenium (II) polypyridyl complexes employing commercially available CF-based columns (Larihc CF6-P, CF6-RN, and CF7-DMP, AZYP, LLC, Arlington, TX, USA) in PO mode [46]. Aromatic derivatives on the selector were found to be essential to obtain enantioselectivity, indicating the importance of π–π interactions in chiral recognition.

An HPLC-based method was developed for the separation of methionine enantiomers applying CF-based CSPs in PO mode [47]. The optimized method using isopropyl carbamate CF-6 bonded onto 5 µm silica particles was validated and successfully applied for the quantitative determination of the enantiomers of methionine in a dietary supplement sample. A study was conducted for the enantioseparation of phenylisoserine analogs with commercially available columns based on CFs (Larihc CF6-P, CF6-RN, CF7-DMP) [48]. Applying *n*-hexane/alcohol eluent systems, NP behavior was typically observed, where the nature of the alcohol modifier exerted a considerable effect both on retention and selectivity, while under PI conditions, the importance of ionic interactions was confirmed.

HPLC and SFC methods were presented for the enantiomeric separations of 21 arylketones [49]. The HPLC generally resulted in higher resolutions for the CF-based columns, applied under NP conditions than utilizing SFC conditions. Based on qualitative measures of structures, structure–separation relationship analysis was performed and an optimized structure for the analyte to be separated was hypothesized. New derivatives of CF6 were prepared by introducing aromatic moieties with electron-withdrawing (chloro and nitro) and electron-donating (methyl) groups [50]. The new columns evaluated under NP conditions showed improved enantioselectivities over the commercially available columns in several cases. The (chloromethyl)phenyl derivatives offered the best enantioresolutions for the studied diverse set of analytes.

In a screening study, the enantioseparation capability of Larihc CF6-P was evaluated and compared to six polysaccharide-based CSPs applying the NP mode with *n*-heptane/ethanol or PO mode with acetonitrile/methanol or 2-propanol eluent systems [51]. The CF-based CSP demonstrated the highest success rate in the separation of 39 nonderivatized chiral primary amines in PO mode. Three CF-based CSPs (Larihc CF6-P, CF6-RN, and CF7-DMP) were evaluated in NP mode with a set of fifteen 2-naphthol-derived atropisomers [52]. CF7-DMP with π-rich phenyl substituents and several residual fructose hydroxyl groups was found to be the most effective CSP, and the importance of steric hindrance and host–guest interaction was also evidenced. The enantiorecognition ability of the same three CSPs was compared in NP mode with 11 racemic analogs of the naturally occurring indole phytoalexyn [53]. CF6-RN promoting π–π and dipolar interactions offered the broadest selectivity. All studied analogs could be at least partially separated with this CSP. The presence of an electron-withdrawing group on the benzene ring of the analytes was found to enhance the enantioselectivity and resolution with reduced retention times.

The effects of mobile phase composition on the enantioseparation of nine analogs of spirobrassinin were evaluated in NP mode [54]. Among the commercially available Larihc columns, that derivatized with the *R*-naphthylethyl carbamate moiety provided the best separations for the studied analogs due to stronger π–π interactions. Larihc CF6-P was applied for the separation of three pentahelicene derivative enantiomers in NP mode [55]. Utilizing the developed dynamic HPLC method, the interconversion energy barriers between the helicene enantiomers were determined. Larihc CF6-P and CF7-DMP were utilized for monitoring the racemization of five biaryl atropisomers [56]. In the thermal stability study, the CF-based CSPs applied under NP conditions provided efficient separations for the atropisomers.

Among other (amylose, and cyclodextrin-based) CSPs, Larihc CF6-P was tested in the separation of panthenol enantiomers [57,58]. With CF6 modified with isopropylcarbamate used under NP conditions, only partial resolution could be achieved for the enantiomers of panthenol, probably due to the disrupted internal H bondings. The chromatographic behavior of deazapurine nucleosides employing bare silica, and amide- and CF6-based stationary phases was studied under HILIC conditions [59]. The Frulic-N column (AZYP, LLC, Arlington, TX, USA) provided ideal peak symmetries for all analytes under all tested conditions. HPLC-based methods were developed for the analysis of phenylalanine enantiomers testing cyclodextrin-, macrocyclic glycopeptide-, and CF6-based columns in RP, NP, and PO separation modes [60]. The CF6 CSP functionalized with isopropylcarbamate showed high efficiency in NP mode, but with relatively long retention times for the Phe enantiomers.

## 6. Enantioseparations Achieved with Cyclofructans Bonded on Ultra-High-Performance Particles

Achieving shorter analysis times and higher efficiencies has always been in the focus of HPLC developments. To carry out fast separations, the column length must be reduced, while the linear velocity must be increased. To preserve theoretical plates, the plate height must significantly be decreased. Developments in particle technology resulted in the availability of sub-2 µm particles, which offer narrower peaks at the expense of higher pressure. Utilizing nonporous and porous particles has been the two major ways to prepare spherical silica particles. However, in the 1960s, the concept of core–shell particles (originally named pellicular particles) was introduced by Horváth et al. [61]. The concept, together with a marked reduction in the particle size, resulted in frequent applications of the core–shell particles in achiral chromatography up until now.

To enhance the efficiency of the CF-based “traditional” CSPs, i.e., utilizing fully porous particles of 5 µm, Armstrong et al. bonded native [62] and isopropylcarbamate-functionalized [63] CF6 to SP (2.7 µm) and FP (3 µm) particles. The prepared columns were evaluated under HILIC, NP, or PO conditions, and the advantages of columns packed with SP particles in analysis time, efficiency, and selectivity were demonstrated. Thanks to further developments, these columns have recently become commercially available. In the following, we briefly discuss the results (summarized in Table 4) obtained with CF-based SP particles published since 2015.

Enantiomeric separations on the order of a few seconds with a broad range of analyte types were demonstrated with macrocyclic antibiotics, hydroxypropyl *β*-cyclodextrin, and CF6- and CF7-based CSPs [11]. The columns packed with 2.7 µm SP particles offered a three-to-four-fold increase in efficiencies compared to 5 µm FP particles, while the CF7-DMP SP-based column provided the smallest reduced plate height reported for any CSP. The ultrafast chiral separations with SP particles were found to be feasible under NP, RP, and PO modes. The same pool of CSPs based on SP particles was applied for the separation of fluorine-containing active pharmaceutical ingredients from their desfluorinated impurities under RP and PO conditions [64]. Most of the separations were achieved in under a minute with resolutions ranging from 1.2 to 4.3 and plate numbers between 45.000 and 70.000 plates/m.

New bonding chemistry using multiple attachment technology was applied to prepare hydrolytically stable CF6-based CSPs [65]. The benzoic acid-modified CF6 employed under HILIC conditions was characterized chromatographically, using acidic, neutral, and basic drugs. The new CSP showed different selectivities and higher efficiencies than carboxylate HILIC phases reported previously, and an improved hydrolytic stability compared to a silica column.

Four commercially available CSPs, all based on 2.7 µm SP particles (VancoShell, NicoShell, CDShell-RSP, and LarihcShell-P, AZYP, LLC, Arlington, TX, USA) were utilized for the enantioseparation of 150 chiral amines of pharmaceutical or toxicological relevance [66]. In this comprehensive study, CSPs were shown to work in a complementary fashion, and the CF6-based LarihcShell-P applied in PO mode was found to be the most effective CSP for separating primary amines. In another study among other commercially available columns, LarihcShell-P was also tested [67]. The CF6-based column employed under NP conditions showed a limited capability for the enantioresolution of different pesticides, namely, only three of the studied compounds could be enantioseparated. A comparative study was conducted to explore the relative merits of SP particle-based columns applied under SFC conditions, where one of the four core–shell CSPs was the LarihcShell-P [68]. Van Deemter curves were measured and evaluated to document the differences induced by the change in the morphology of the support particles. The isopropyl-derivatized CF6-based column could efficiently separate the studied primary amines and amino acids. The positive effects of the replacement of methanol with azeotropic ethanol as a less expensive and easy-to-recycle co-solvent were demonstrated in an SFC study [69]. One of the studied columns was the CF6-based LarihcShell, offering excellent enantioseparations for the studied primary amines and amino acids.

An LC–MS/MS method was developed for the quantitative determination of enantiomers of verapamil in rat plasma [70]. For the enantiorecognition, three CF-based columns (Larihc CF6-RN, CF7-DMP, and CF6-P) were tested in NP, RP, and PO modes. The separation of the enantiomers was achieved with the isopropyl-functionalized CF6-based column. The method was optimized, validated, and successfully applied in a pharmacokinetic study.

## 7. Conclusions

Regarding the synthesis of new chiral selectors and CSPs, method developments, and chiral/achiral recognition studies, this brief overview underlined the important role of CSPs based on crown ethers and cyclofructans in chiral chromatography. However, the widespread use of these kinds of CSPs is limited, because they are suitable only for the separation of compounds containing protonated amine functional group(s).

## Figures and Tables

**Table 1:** Common chiral stationary phases, their selectors, and the most important interactions for chiral recognition.

CSP Type		Selector	Most Important Interactions
1.	ligand exchange	amino acid–metal complex	complex formation
2.	protein-based	natural proteins	H-bridge, ionic, van der Waals forces, *π*–*π*
3–5.	cavity-type	cyclodextrins, cyclofructans, chiral crown ethers	complex formation, ionic, hydrophobic, H-bridge, van der Waals forces, steric, *π*–*π*
6.	donor–acceptor (*Pirkle*-type)	*π*-acidic, *π*-basic compounds	H–bridge, *π*–*π*, dipole–dipole, steric
7.	synthetic polymers	polyacrylamides, polymethacrylates, polyisocyanides, etc.	H-bridge, *π*–*π*, steric
8.	molecularly imprinted polymers	selective sorbents (e.g., organic copolymers)	steric, H-bridge, *π*–*π*
9.	ion exchanger	anion and cation exchangers, zwitterionic compounds	ionic, H-bridge, *π*–*π*, steric
10.	macrocyclic antibiotics	macrocyclic glycopeptides	ionic, H-bridge, *π*–*π*, hydrophobic, steric
11.	derivatized polysaccharides	derivatized cellulose and amylose	H-bridge, *π–π,* van der Waals forces

**Table 2:** High-performance liquid chromatographic enantioseparations on crown-ether-based chiral stationary phases ^1^.

Chiral Analytes	Selector	Column Characteristics Trademark Particle Size	The Most Effective Mobile Phase (*v*/*v*/*v*/*v*)	Mode	Ref.
warfarin, metoprolol, propranolol, ibuprofen, proglumide, indapamide, etc.	aza-15-crown-5-capped methylcalix [4] resorcinarene bonded to 2-hydroxy-propoxy-propylsilyl silica	15C5-MCR-HPS, 5 μm	MeOH/H_2_O 40/60; 10/90 2-propanol/hexane 3/97 MeCN/H_2_O 10/90; 20/80; 30/70 MeCN/phosphate buffer (pH 8.0) 10/90; 20/80	NPM RPM	[26]
arylalkylamines, phenylalanine methyl- and benzyl esters, phenylglycine, methyl-and benzyl esters	pyridine-18-crown-6 ether-based CSP	(*S*,*S*)-CSP-1, 5 μm	MeOH/MeCN 90/10 MeOH/MeCN 85/15 MeOH/MeCN 80/20 MeOH/MeCN/TEA/FA 80/20/0.2/0.2 MeOH/MeCN/TEA/FA 80/20/0.1/0.1	POM PIM	[27]
arylalkylamines, α-amino acid esters	dimethyl-substituted acridino-18-crown-6 and acridino-21-crown-7 containing carboxyl group at position 9	(*S*,*S*)-CSP-8, 5 μm (*S*,*S*)-CSP-12, 5 μm	MeCN/25 mM aq. NH_4_OAc 20/80 MeCN/40 mM aq. NH_4_OAc 20/80	RPM	[28]
primary amines including amino acids	crown ether moiety modified by (1*S*,2*S*)-2-aminocyclohexyl phenyl-carbamate and connected to quinine or quinidine through carbamate at C-9 position	CSP-1–CSP-4, 5 μm	EtOH/FA/DEA 100/01/0.1 MeOH/FA/DEA 100/0.1/0.1 MeCN/H_2_O 90/10 + 5 mM LiClO_4_ MeCN/H_2_O 85/15 + 5 mM LiClO_4_	PIM RPM	[29]
acyclovir, valacyclovir and its analogs	3,3′-diphenyl-1,1′-binaph-thyl-20-crown-6	CSP-1, 5 μm, with residual silanol CSP-2, 5 μm, *n*-octyl protected silanols	0.05–0.15 mM *aq.* HClO_4_/MeCN 70/30 0.10 mM *aq.* TFA/MeCN 70/30 0.05–0.15 mM *aq.* HClO_4_/MeOH 70/30 0.05–0.15 mM *aq.* HClO_4_/EtOH 70/30	RPM	[30]
20 α-amino acids	(*S*)-(+)-3,3′-phenyl-1,1′-binaphthyl-18-crown-6	CROWNPAK CR-I (+), 5 μm	A: MeCN/H_2_O/TFA 50/50/0.5 B: MeCN/TFA 100/0.5 A/B 50/50	RPM LC-MS	[31]
20 pairs of α-amino acids in black vinegar	(*S*)-(+)- and (*R*)-(−)-3,3′-diphenyl-1,1′-binaphthyl-20-crown-6	CROWNPAK CR-I (+), 5 μm CROWNPAK CR-I (−), 5 μm	MeCN/EtOH/H_2_O/TFA 85/15/5/0.5	RPM LC-MS	[32]
18 pairs of α-amino acids	(*S*)-(+)-3,3′-phenyl-1,1′-binaphthyl-20-crown-6	CROWNPAK CR-I-(+), 5 μm CROWNPAK CRI-(+), 3 μm	MeCN/H_2_O/TFA 96/4/0.5 MeCN/EtOH/H_2_O/TFA 80/15/5/0.5	RPM LC-MS	[33]
substituted aniline position isomers	(*R*)-(−)-3,3′-diphenyl-1,1′-binaphthyl-20-crown-6	CROWNPAK CR-I (−), 5 μm	H_2_O/MeCN (70/30) + 20 mM HClO_4_ H_2_O/MeCN (10/90) + 20 mM HClO_4_ *n*-hexane/EtOH/H_2_O/TFA 100/100/4/1	RPM NPM	[34]
d,l-phenylseptin peptides	(*S*)-(+)- and (*R*)-(−)-3,3′-phenyl-1,1′-binaphthyl-18-crown-6	CROWNPAK CR-I (+), 5 μm CROWNPAK CR-I (−), 5 μm	aq. HClO_4_ pH 1.0/ /MeCN/MeOH 15/25/60	RPM	[35]
Tyr-Arg-Phe-Lys-NH_2_	(*S*)-(+)-3,3′-diphenyl-1,1′-binaph- thyl-20-crown-6 (+)-18-crown-6)-2,3,11,12-tetracarboxylic acid	CROWNPAK CR-I-(+), 5 μm ChiroSil RCA (+), 5 μm	50 mM aq. HClO_4_/MeCN (5–85)/(95–15) 50 mM aq. HClO_4_/MeCN (90–80)/(10–20)	RPM	[36]
amlodipine	(*S*)-(+)-3,3′-diphenyl-1,1′-binaphthyl-20-crown-6	CROWNPAK CR (+), 5 μm	70% aq. HClO_4_ (pH 2.0)/ /MeOH 95/5	RPM	[37]
21 pairs of α-amino acids	(*R*)-3,3′-dibromo (or dichloro or diiodo)phenyl-1,1′-binaphthyl-20-crown-6	5 μm	10 mM aq. HClO_4_ (pH 2)	RPM	[38]
DPP-4 inhibitors: alogliptin, linagliptin, saxagliptin	(*S*)-(+)-3,3′-diphenyl-1,1′-binaphthyl-20-crown-6	CROWNPAK CR (+), 5 μm	aq. HClO_4_ (pH 1.0; 1.5)/ /MeOH 80/20 aq. HClO_4_ (pH 1.0; 1.5)/ /EtOH 80/20 aq. HClO_4_ (pH 1.0)/ /2-propanol 80/20 aq. HClO_4_ (pH 1.0; 1.5)/ /MeCN 80/20 aq. HClO_4_ (pH 1.0; 1.5)/THF 80/20	RPM	[39]
18 pairs of α-amino acids	(*S*)-(+)-3,3′-diphenyl-1,1′-binaphthyl-18-crown-6	CROWNPAK CR-I (+), 5 μm	EtOH/H_2_O/TFA 95/5/0.8	SFC MS	[40]
quinolones: primaquine, lomefloxacin, tafenoquine, flumequine, ofloxacin	(+)-18-crown-6–2,3,11,12-tetracarboxylic acid	(+)-Crownpak, 5 μm	MeCN/H_2_O 80/20 + 10 mM H_2_SO_4_ + 10 mM NH_4_OAc MeCN/H_2_O 80/20 + 20 mM HClO_4_ EtOH/H_2_O 80/20 + 20 mM HClO_4_	RPM	[41]
thyroxine enantiomers	(+)-18-crown-6–2,3,11,12-tetracarboxylic acid	ChiroSil RCA (+), 5-μm	MeOH/H_2_O/TFA 80/20/0.07	UPLC-MS	[42]
proline, pipecolic acid derivatives	(+)-18-crown-6–2,3,11,12-tetracarboxylic acid	CSP-1, 5 μm; free N-H group on silica CSP-2, 5 μm; methylated N-H group on silica	MeOH/MeCN/AcOH/TEA 30/70/0.2/0.2 MeOH/MeCN/AcOH/TEA 30/70/0.1/0.3 MeOH/MeCN/AcOH/TEA 50/50/0.2/0.2	PIM	[43]
methoxyphenamine and its analogs	(+)-18-crown-6–2,3,11,12-tetracarboxylic acid	CSP-1, 5 μm; free N-H group on silica CSP-2, 5 μm; methylated N-H group on silica	MeOH/MeCN/AcOH/TEA 50/50/0.1/0.5	PIM	[44]

^1^ Abbreviations: RPM, reversed-phase mode; NPM, normal-phase mode; PIM, polar ionic mode; POM, polar organic mode.

**Table 3:** High-performance liquid chromatographic enantioseparations on cyclofructan-based chiral stationary phases ^1^.

Chiral Analytes	Selector	Column Characteristics Trademark Particle Size	The most Effective Mobile Phase (*v*/*v*/*v*/*v*)	Mode	Ref.
34 acids, warfarin, bi-2-naphthol, furoin, phenylglycinol, phensuximide, temazepam, etc.	CF-6 derivatized with propylimidazole, methylbenzimidazole, dimethylaminopropyl, pyridine, dimethylaminophenyl	SP-CF6-IM, 5 μm SP-CF6-BIM, 5 μm SP-CF6-AP, 5 μm SP-CF6-PY, 5 μm SP-CF6-DMAP, 5 μm	*n*-heptane/EtOH/TFA 70/30/0.1; 80/20/0.1; 90/10/0.1 and 95/5/0.1 MeCN/MeOH/AcOH/TEA 60/40/0.3/0.2; 80/20/0.3/0.2; 98/2/0.3/0.2	NPM POM	[45]
21 ruthenium (II) polypyridyl complexes	CF6-isopropylcarbamate, CF6-*R*-1-1-naphthyl-ethyl-carbamate, CF7-dimethyl-phenyl- carbamate	LARIHC-CF6-P, 5 μm LARIHC-CF6-RN, 5 μm LARIHC-CF7-DMP, 5 μm	MeCN/MeOH/AcOH/TEA 30/70/1.6/4.0 MeOH/AcOH/TEA 100/1.6/4.0; 100/1.6/2.4 MeCN/MeOH + 0.05 M N(CH_3_)_4_NO_3_	POM	[46]
methionine	CF6-isopropylcarbamate	IP-CF6, 5 μm	MeOH/MeCN/AcOH/TEA 75/25/0.3/0.2	POM	[47]
phenylisoserine derivatives	CF6-isopropylcarbamate, CF6-*R*-1-1-naphthyl-ethyl-carbamate	LARIHC-CF6-P, 5 μm LARIHC-CF6-RN, 5 μm	*n*-hexane/2-PrOH/TFA 50/50/0.1, 30/70/0.1; 10/90/0.1 *n*-hexane/2-PrOH/HClO_4_ 10/90/0.1 MeOH/MeCN/TFA/TEA 10/90/0.3/0.2	NPM PIM	[48]
21 α-aryl ketones	CF6-isopropylcarbamate, CF6-*R*-1-1-naphthyl-ethyl-carbamate, CF7-dimethyl-phenyl-carbamate	LARIHC-CF6-P, 5 μm LARIHC-CF6-RN, 5 μm LARIHC-CF7-DMP, 5 μm	*n*-heptane/EtOH 90/10, 95/5, 98/2 *n*-heptane/2-PrOH 98/2 CO_2_/EtOH 95/5 or 98/2	NPM SFC	[49]
BINOL, BINAM, thalidomide, warfarin, furoin, Troger’s base, *trans*-stilbene oxide, etc.	CF6 derivatized with 10 aromatic chloro and nitro moieties	4C3MP, 3C4MP, 4CP, 3,5-DCP, 3,4DCP, 3CP, 4C2NP, 4C3NP, 4MP, 4C2MP	*n*-heptane/EtOH 95/5	NPM	[50]
primary amines	CF6-isopropylcarbamate	LARIHC-CF6-P, 5 μm	*n*-heptane/EtOH/TFA/TEA 60/40/03/02 MeCN/MeOH/TFA/TEA 90/10/0.3/0.2; 60/40/0.3/0.2	NPM POM	[51]
2-naphthol atropisomers	CF6-isopropylcarbamate, CF6-*R*-1-1-naphthyl-ethyl-carbamate, CF7-dimethyl-phenyl-carbamate	LARIHC-CF6-P, 5 μm LARIHC-CF6-RN, 5 μm LARIHC-CF7-DMP, 5 μm	*n*-heptane/EtOH 95/5 *n*-heptane/2-PrOH 95/5; 90/10 n-heptane/BuOH 95/5	NPM	[52]
indole phytoalexin analogs	CF6-isopropylcarbamate, CF6-*R*-1-1-naphthyl-ethyl- carbamate, CF7-dimethyl-phenyl- carbamate	LARIHC-CF6-P, 5 μm LARIHC-CF6-RN, 5 μm LARIHC-CF7-DMP, 5 μm	*n*-hexane/2-propanol/TFA 90/10/0.1 *n*-hexane/2-propanol/TEA 98/2/0.1; 95/5/0.1; 90/10/0.1 *n*-hexane/EtOH/TFA 90/10/0.1; 80/20/0.1; 70/30/0.1 *n*-hexane/EtOH/TEA 98/2/0.1; 95/5/0.1; 90/10/0.1;	NPM	[53]
spirobrassinin analogs	CF6-isopropylcarbamate, CF6-*R*-1-1-naphthyl-ethyl-carbamate, CF7-dimethyl-phenyl-carbamate	LARIHC-CF6-P, 5 μm LARIHC-CF6-RN, 5 μm LARIHC-CF7-DMP, 5 μm	*n*-hexane/2-propanol 90/10; 80/20 *n*-hexane/EtOH 90/10; 80/20 *n*-hexane/2-propanol/TFA 80/20/0.1; 70/30/0.1 *n*-hexane/2-propanol/TFA/TEA 80/20/0.3/0.2	NPM	[54]
pentahelicene-derivatives	CF6-isopropylcarbamate	LARIHC-CF6-P, 5 μm	*n*-heptane/MeOH 99.9/0.1	NPM	[55]
biaryl atropisomers: BINOL, BINAM, NOBIN, Phenap, trichlophbin	CF6-isopropylcarbamate, CF7-dimethyl-phenyl-carbamate	LARIHC-CF6-P, 5 μm LARIHC-CF7-DMP, 5 μm	*n*-heptane/EtOH 90/10 *n*-heptane/EtOH/butylamine 90/10/0.1	NPM	[56]
panthenol	CF6-isopropylcarbamate	LARIHC-CF6-P, 5 μm	*n*-hexane/2-propanol/TFA/TEA 90/10/0.2/0.3; 75/25/0.1/0.1; 75/25/0.2/0.2; 75/25/0.1/0.2; 75/25/0.2/0.1; 50/50/0.1/0.1; 25/75/0.1/0.1	NPM	[57]
panthenol	CF6-isopropylcarbamate	LARIHC-CF6-P, 5 μm	*n*-hexane/2-propanol 75/25 *n*-hexane/2-propanol/TFA/TEA 75/25/0.2/0.1	NPM	[58]
7-deazopurine-(pyrrolo[2,3-*d*]-pyrimidine) nucleosides	native cyclofructan	FRULIC-N, 5 μm	MeCN/aq. 10–50 mM NH_4_OAc (pH 4.7 or 7.5) 97/3; 95/5; 90/10; 80/20	HILIC	[59]
phenylalanine	CF6-isopropylcarbamate	IP-CF6, 5 μm	MeOH/MeCN/AcOH/TEA 30/70/0.3/0.2 *n*-hexane/EtOH/TFA 60/40/0.1	PIM NPM	[60]

^1^ Abbreviations: CF6, cyclofructan-6; CF7, cyclofructan-7; RPM, reversed-phase mode; NPM, normal-phase mode; PIM, polar ionic mode; POM, polar organic mode; HILIC, hydrophilic interaction liquid chromatography; SFC, supercritical fluid chromatography; BINOL, 1,1′-binaphthyl-2,2′-diol; BINAM, 1,1′-binaphthyl-2,2′-diamine; NOBIN, 2′-amino-1,1′-binaphthyl-2-ol; Phenap, 2-aminophenyl-1,1′-naphthalen-2-amine; Trichlophbin, 2′-amino-3′,4′,5′-trichlorophenyl-1,1′-naphthalen-2-ol.

**Table 4:** Ultra-high-performance liquid chromatographic enantioseparations on derivatized cyclofructan analogs immobilized on 2.7 μm superficially porous particles ^1^.

Chiral Analytes	Selector	Trademark	The Most Effective Mobile Phase (*v*/*v*/*v*/*v*)	Mode	Ref.
amines, alcohols, norphenylephrine, normetanephrine, tryptophanol, 1-amino-2-indanol, (*R*)-(+)-2′-amino-1,1′-bi-naphthalen-2-ol, etc.	CF6-isopropyl-carbamate, CF7-dimethyl-phenyl-carbamate	CF6-P, CF7-DMP	MeOH/MeCN/TFA/TEA 20/80/0.3/0.2 MeOH/MeCN/TFA/TEA 30/70/0.3/0.2 MeOH/MeCN/TFA/TEA 8/92/0.3/0.2 *n*-heptane/EtOH 90/10; 95/5; 98/2 *n*-heptane/EtOH/TFA 70/30/0.1	PIM NPM	[11]
fluorinated, desfluorinated analytes: ofloxacin, ciprofloxacin, voriconazole, atorvastatin	CF6-isopropyl-carbamate	CF6-P	MeOH/MeCN/TFA/TEA 10/90/0.3/0.2 MeOH/MeCN/TFA/TEA 5/95/0.3/0.2 MeOH/MeCN/TFA/TEA 2/98/0.3/0.2	PIM	[64]
salicylic acid analogs, *β*-blockers, nucleic acids	CF6-benzoic acid	CF6-benzoic acid	20 mM NH_4_OAc pH 4.2/MeCN 25/75 H_2_O/MeCN 15/85	HILIC	[65]
pharmaceuticals, stimulants, reagents, amino acids and derivatives	CF6-isopropyl-carbamate	LarihcShell-P	MeOH/MeCN/AcOH/TEA (10–90)/(90–10)/0.3/0.2 MeOH/MeCN/TFA/TEA (5–10)/(95–90)/0.3/0.2 *n*-heptane/EtOH/TFA/TEA 90/10/0.3/0.2	PIM NPM	[66]
closantel, famoxadone, fipronil	CF6-isopropyl carbamate	LarihcShell-P	*n*-hexane/2-propanol 95/5 *n*-heptane/2-propanol/TFA/TEA 90/10/0.3/0.2	NPM	[67]
primary amines, norephedrine, normetanephrine, norphenylephrine, octopamine, tryptophan amide, Trp, Phe, etc.	CF6-isopropyl-carbamate	LarihcShell-P	CO_2_/MeOH/TEA/TFA 75/25/0.2/0.3 CO_2_/MeOH/TEA/TFA 80/20/0.2/0.3	SFC	[68]
Trp, 1,2,2-triphenylethylamine, 2-chloroindan-1-ylamine	CF6-isopropyl-carbamate	LarihcShell-P	CO_2_/MeOH/TEA/TFA 75/25/0.2/0.3; 80/20/0.2/0.3; 85/15/0.2/0.3 CO_2_/EtOH*/TEA/TFA 75/25/0.2/0.3; 80/20/0.2/0.3; 85/15/0.2/0.3	SFC	[69]
verapamil in rat plasma	CF6-isopropyl-carbamate	LarihcShell-P	MeCN/10 mM ammonium formate/TFA 99.8/0.1/0.1	RPM	[70]

^1^ Abbreviations: CF6, cyclofructan6; CF7, cyclofructan7; EtOH*, “190 proof” EtOH; SPP, superficially porous particles; FPP, fully porous particles; RPM, reversed-phase mode; NPM, normal-phase mode; PIM, polar ionic mode; POM, polar organic mode; HILIC, hydrophilic interaction liquid chromatography; SFC, supercritical fluid chromatography.

## Data Availability

Not applicable.
